# Point-of-care diagnosis and monitoring of fibrinolysis resistance in the critically ill: results from a feasibility study

**DOI:** 10.1186/s13054-023-04329-5

**Published:** 2023-02-10

**Authors:** Lucy A. Coupland, David J. Rabbolini, Jonathan G. Schoenecker, Philip J. Crispin, Jennene J. Miller, Tony Ghent, Robert L. Medcalf, Anders E. Aneman

**Affiliations:** 1 Intensive Care Unit, Liverpool Hospital, Liverpool, Australia; 2 Ingham Institute for Applied Medical Research, 1 Campbell St, Liverpool, NSW 2170 Australia; 3 Kolling Institute of Medical Research, Faculty of Medicine and Health, University of Sydney, Sydney, Australia; 4 Oxford Haemophilia and Thrombosis Centre, Oxford University Hospitals NHS Foundation Trust, Oxford, UK; 5 Department of Orthopaedics and Pharmacology, Vanderbilt University Medical Center, Nashville, TN USA; 6 Haematology Department, The Canberra Hospital, Canberra, Australia; 7 The Australian National University Medical School, Canberra, Australia; 8 Intensive Care Unit, Gold Coast University Hospital, South Port, Australia; 9 Australian Centre for Blood Diseases, Monash University, Melbourne, Australia

**Keywords:** Fibrinolysis resistance, Fibrinolytic shutdown, Fibrinolysis, Viscoelastic testing, Point-of-care testing, Tissue plasminogen activator, Plasminogen, Alteplase, Personalised medicine

## Abstract

**Background:**

Fibrinolysisis is essential for vascular blood flow maintenance and is triggered by endothelial and platelet release of tissue plasminogen activator (t-PA). In certain critical conditions, e.g. sepsis, acute respiratory failure (ARF) and trauma, the fibrinolytic response is reduced and may lead to widespread thrombosis and multi-organ failure. The mechanisms underpinning fibrinolysis resistance include reduced t-PA expression and/or release, reduced t-PA and/or plasmin effect due to elevated inhibitor levels, increased consumption and/or clearance. This study in critically ill patients with fibrinolysis resistance aimed to evaluate the ability of t-PA and plasminogen supplementation to restore fibrinolysis with assessment using point-of-care ClotPro viscoelastic testing (VET).

**Methods:**

In prospective, observational studies, whole-blood ClotPro VET evaluation was carried out in 105 critically ill patients. In 32 of 58 patients identified as fibrinolysis-resistant (clot lysis time > 300 s on the TPA-test: tissue factor activated coagulation with t-PA accelerated fibrinolysis), consecutive experimental whole-blood VET was carried out with repeat TPA-tests spiked with additional t-PA and/or plasminogen and the effect on lysis time determined. In an interventional study in a patient with ARF and fibrinolysis resistance, the impact of a 24 h intravenous low-dose alteplase infusion on coagulation and fibrinolysis was prospectively monitored using standard ClotPro VET.

**Results:**

Distinct response groups emerged in the ex vivo experimental VET, with increased fibrinolysis observed following supplementation with (i) t-PA only or (ii) plasminogen and t-PA. A baseline TPA-test lysis time of > 1000 s was associated with the latter group. In the interventional study, a gradual reduction (25%) in serial TPA-test lysis times was observed during the 24 h low-dose alteplase infusion.

**Conclusions:**

ClotPro viscoelastic testing, the associated TPA-test and the novel experimental assays may be utilised to (i) investigate the potential mechanisms of fibrinolysis resistance, (ii) guide corrective treatment and (iii) monitor in real-time the treatment effect. Such a precision medicine and personalised treatment approach to the management of fibrinolysis resistance has the potential to increase treatment benefit, while minimising adverse events in critically ill patients.

*Trial registration*: VETtiPAT-ARF, a clinical trial evaluating ClotPro-guided t-PA (alteplase) administration in fibrinolysis-resistant patients with ARF, is ongoing (ClinicalTrials.gov NCT05540834; retrospectively registered September 15th 2022).

**Supplementary Information:**

The online version contains supplementary material available at 10.1186/s13054-023-04329-5.

## Introduction

Fibrinolysis, the breakdown of fibrin, is a normal process that maintains blood flow within the vasculature. Due to the progressive decrease in blood vessel diameters, fibrin deposits and small thrombi are filtered into smaller blood vessels, and here, fibrinolysis is required for dissolving such thrombi. Fibrinolysis is triggered following the release of tissue plasminogen activator (t-PA) by platelets and the endothelium, which converts plasminogen to plasmin, that then cleaves fibrin. This process is modulated by the concentrations of t-PA and plasminogen, as well as several inhibitors that target these proteins or modify fibrin to prevent its degradation [1, 2].

Severe infection and tissue injury induce dynamic and interconnected responses in the inflammatory, coagulation and fibrinolytic systems [3, 4]. Significant perturbations in fibrinolysis may result in severe haemorrhage (hyperfibrinolysis) or macro- or micro-thrombosis potentially leading to end-organ damage (fibrinolysis resistance) [5–9], with several studies providing evidence that initial hyperfibrinolysis transitions to fibrinolysis resistance due to factor consumption [9, 10]. In critical conditions, such as sepsis, COVID-19 and non-COVID-19 acute respiratory failure (ARF) and extensive trauma, fibrinolysis resistance has been associated with elevated levels of plasminogen activator inhibitor-1 (PAI-1) [6–8, 11–13]. In another study in COVID-19 disease, elevated levels of both PAI-1 and t-PA levels were reported, with hyperfibrinolysis associated with high t-PA levels and a poor outcome [14]. A consumptive mechanism for the fibrinolysis resistance seen in severe COVID-19 disease was postulated by Medcalf et al. [15]. The authors propose that levels of fibrin and necrotic material, generated during the infective/inflammatory process in the lungs, consume plasmin leading to pathway exhaustion from deficiency of factors such as plasminogen. Furthermore, that fibrinolysis can be restored by enhancing plasmin formation either via administration of t-PA or its substrate, plasminogen [15]. This hypothesis is supported by a study demonstrating that low plasminogen levels are associated with increased mortality in severe COVID-19 disease [16] and by the apparent beneficial effects of direct fibrinolytic approaches using either t-PA [17] or plasminogen [18].

Fibrinolysis is a highly localised process, which only happens in the presence of t-PA. This dynamic process is not assessed by any standard coagulation assay. Approaches to evaluate the fibrinolytic response to added t-PA have been repeatedly investigated in recent years, both in plasma and in whole blood. As platelets also play a role in fibrinolysis [19, 20], functional tests for the fibrinolytic response to t-PA in fresh whole blood, rather than platelet poor plasma, would seem appropriate. Whole blood-based approaches for the functional assessment of the fibrinolytic response to t-PA have usually been performed using viscoelastic testing (VET), a method that allows for a comprehensive assessment of coagulation and fibrinolysis by a continuous assessment of blood clot firmness [21]. Approaches to perform VET with added t-PA have been described since 2006 [20, 22–26]; however, only recently has a standardised assay been commercially available and approved for diagnostic use. In the ClotPro TPA-test, coagulation is triggered by a combination of recombinant tissue factor and calcium chloride, and fibrinolysis is triggered by a high dose of recombinant t-PA. The assay has been used in various studies that evaluated the decrease of fibrinolysis in critically ill patients following COVID-19 infection [27–30] with several studies correlating VET parameters with the severity of COVID-19 disease [31–35].

This study in critically ill patients with fibrinolysis resistance aimed to evaluate the ability of t-PA and plasminogen supplementation to restore fibrinolysis with assessment using point-of-care (POC) ClotPro viscoelastic testing (VET). We performed prospective observational and interventional point-of-care experiments using ClotPro and its TPA-test in critically ill patients to initially select individuals with fibrinolysis resistance. These results are presented for COVID-19 patients, non-COVID-19 patients and healthy controls to enable a comparison to be made of the severity of fibrinolysis resistance present, given the prominence in the recent literature on fibrinolysis resistance in COVID-19 including novel trials of t-PA and plasminogen administration [17, 18, 36]. Importantly, these trials have not incorporated individualised assessments of fibrinolysis resistance or treatment responses. Consecutive exploratory VET analyses were conducted in a proportion of fibrinolysis-resistant patients with ex vivo spiking of the TPA-test with additional t-PA with/without plasminogen to assess the requirements for restoration of fibrinolysis. In addition, in a fibrinolysis-resistant patient with bacterial pneumonia causing ARF, alteplase (t-PA) was administered over 24 h and standard ClotPro VET was used to regularly monitor the effect on coagulation and fibrinolysis.

We hypothesised, based on the existing literature, that fibrinolysis-resistant patients would display significant variation in the degree of fibrinolytic compromise that could be corrected through supplementation with additional t-PA and/or plasminogen. We also hypothesised that the ClotPro TPA-test would offer rapid point-of-care capability to monitor the effect on coagulation and fibrinolysis of intravenous t-PA administration in hypofibrinolytic patients, thus permitting a personalised dose that maximised benefit while minimising risk.

## Methods

ClotPro® (enicor GmbH, Munich, Germany) was used for viscoelastometry. ClotPro is a new-generation viscoelastometry analyser, which has shown good agreement with the widely used ROTEM delta device [37, 38].

In all individuals, blood was collected into 3.2% buffered sodium citrate tubes and analysed by ClotPro within 30 min of collection using the EX-test®, FIB-test® and TPA-test® (standard Clotpro assays) as per the manufacturer’s instructions. In all three assays, coagulation is activated by recombinant tissue factor, CaCl_2_ is used to recalcify the sample and polybrene is used to block heparin effects. In the FIB-test, the platelet contribution to the clot is blocked by a combination of cytochalasin D (an actin polymerisation inhibitor) and eptifibatide, a small molecular inhibitor of the glycoprotein α2bβ3 receptor (GpIIb/IIIa). In the TPA-test, recombinant t-PA (650 ng/mL) is used to trigger fibrinolysis and the lysis time (LT) is calculated by the associated software that indicates the time taken for the maximum clot firmness to be reduced by 50%. Blood fibrinogen concentrations will influence the volume of the fibrin clot formed and endogenous blood levels of the fibrinolytic proteins, t-PA and plasminogen, and their inhibitors will influence the rate of clot lysis. Standard ClotPro assays were performed by ICU nurses, registrars, residents, consultants and research staff trained on the ClotPro device, with a subset of medical and research staff carrying out the experimental tPA/plasminogen supplementation assays.

**Fig. 1:**
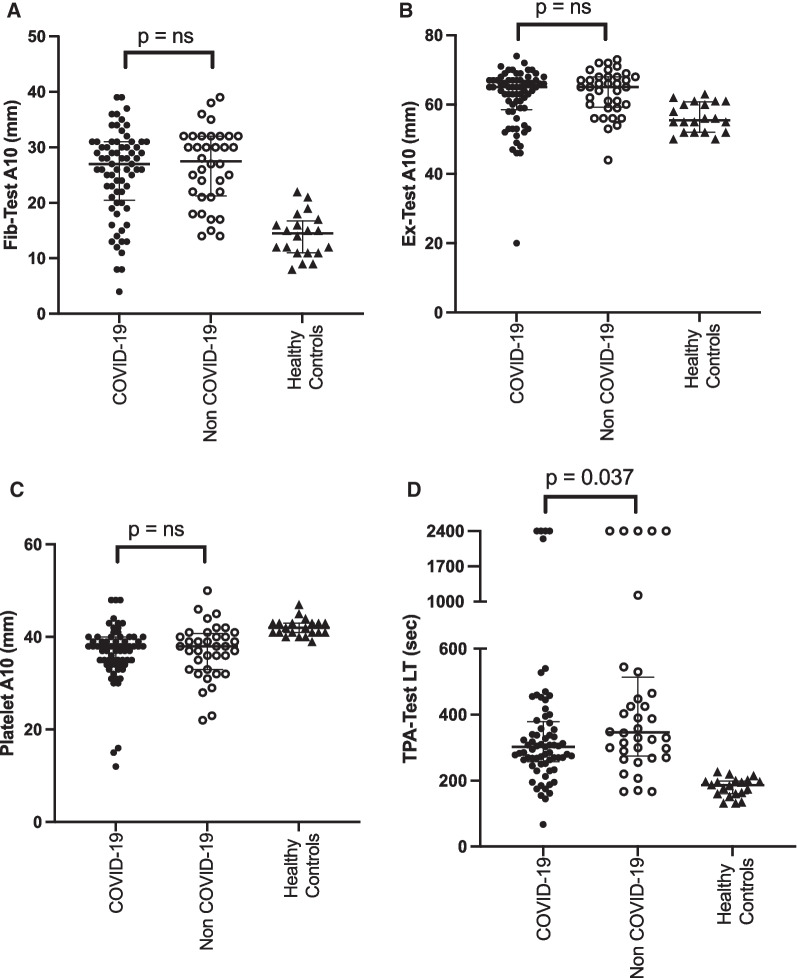
Similar levels of disordered coagulation and fibrinolysis occur in critically ill patients with or without COVID-19 disease. Standard ClotPro analysis was carried out from patients admitted to intensive care (*n* = 105) and the results compared based on the presence of COVID-19, with the results obtained from healthy ICU staff provided as a reference (*n* = 20). **A** FIB-test A10 indicates the fibrin clot amplitude 10 min after the commencement of clot formation stimulated with tissue factor. **B** EX-test A10 represents the fibrin (ogen) and platelet-dependent clot amplitude 10 min after the commencement of clot formation stimulated with tissue factor. **C** Platelet A10 is calculated by subtracting the FIB-test A10 value from the EX-test A10 value and indicates the platelet contribution to the clot. **D** TPA-test LT indicates the time at which 50% of the maximum fibrin clot formed is degraded by recombinant t-PA (650 ng/mL blood). A value of 2400 s indicates no lysis occurred within the timeframe of the assay. Individual data points presented, bars represent median and interquartile ranges. Nonparametric Mann–Whitney test performed between patient groups; ns = nonsignificant *p* value

The manufacturer’s normal ranges were used for the EX-test and FIB-test. The platelet contribution to the clot, platelet A10, was calculated by subtracting the FIB-test A10 value from the EX-test A10 value. For the determination of fibrinolysis resistance, we used TPA-test lysis times of 300 s or greater, this value aligning with the 90th percentile of a blood donor population (*n* = 60; 304 s) [29], and being > 4 standard deviations above the mean LT obtained in our healthy staff controls (*n* = 20; mean 180.4 ± SD 28.6 s) (Fig. 1D).

A total of 105 COVID-19-infected and non-COVID-19-infected patients admitted to the intensive care unit were screened using standard ClotPro assays. Fifty-eight individuals were identified as fibrinolysis-resistant (TPA-test LT > 300 s). Of these 58, 32 were included in the ex vivo supplementation experiments (described below). Availability of reagents and staff trained in the experimental procedures determined which patients were included in these supplementation experiments. Twenty controls (healthy ICU staff members) were also studied to establish normal fibrinolysis parameters (Additional file 1: Fig. S1).

### Ex vivo VET supplementation experiments

Repeat viscoelastometry was consecutively performed on the same blood sample in fibrinolysis-resistant patients with ex vivo spiking of the TPA-test with additional t-PA (650–1300 ng t-PA/mL blood) by passing the blood through 1 or 2 additional TPA-test tips (enicor GmbH, Munich, Germany). Such supplementation would also double the concentrations of tissue factor, CaCl_2_ and polybrene being added to the blood. To study the effect of increased concentrations of these reagent components on coagulation, sub-studies were performed using blood samples from two patients which were passed through 1 and 2 EX-test tips prior to the commencement of the assay. These experiments demonstrated a < 5% difference in the clot amplitude at 10 min (A10), maximum clot firmness (MCF) and lysis index at 30 min (CLI30).

Plasminogen (Hyphen-Biomed, France) was initially added to the ClotPro cup prior to the commencement of the assay using freshly thawed plasminogen reconstituted in deionised water (stock 2 µg/uL) to achieve a final concentration in the ClotPro analysis cup of 59 µg/mL blood, about a third of reported blood concentrations [39]. Subsequently, to streamline the process for point-of-care testing and to enable an increased dose to be tested without diluting the blood, preloaded pipette tips were prepared with lyophilised plasminogen that could be used on the ClotPro automated pipette and that achieved a concentration of 147 µg/mL blood, this value being on the lower end of reported normal blood concentrations (150–200 µg/mL). The blood was drawn up through the plasminogen-loaded tip and placed into the cup with a mixing step, prior to the blood then being drawn up from the cup through the TPA-test tip (containing CaCl_2_) with the usual mixing step. The assay was then commenced after this second step.

### In vivo study

In a fibrinolysis-resistant patient with ARF entered into a clinical trial of VET-guided treatment using low-dose alteplase, the EX-test, FIB-test and TPA-test were repeated regularly throughout the 24-h infusion and one hour after cessation and analyses commenced within 10 min of blood collection.

### Data analysis

The data were analysed using PRISM V9.0 software (GraphPad, CA). Results are reported as medians including the interquartile range within brackets [IQR], since non-normally distributed as per the Shapiro–Wilk test. Comparisons were made using the Mann–Whitney test for non-normally distributed data (D’Agostino and Pearson test) and reported including the median difference and its 95% confidence interval (95% CI). Correlations were analysed by Spearman’s correlation coefficient (rho) and reported including the 95% CI. Two-sided *p*-values are reported for all analyses. Statistical significance was set at a two-sided *p*-value of < 0.05.

## Results

### Standard ClotPro assays

A total of 105 patients were assessed using ClotPro within a median of 1 [1, 2] day of admission to the intensive care unit. Table 1 summarises the characteristics of the patient population studied and divided into groups with or without fibrinolysis resistance and with or without a diagnosis of SARS-CoV2 (COVID-19) infection.

**Table 1:** Patient characteristics

	All patients screened (*n* = 105)	No fibrinolysis resistance (LT < 300) (*n* = 47)	Fibrinolysis resistance (LT > 300) (*n* = 58)	COVID + ve (*n* = 69)**	COVID − ve (*n* = 36)
*Demographic and treatment variables*					
Age	62 [45–71]	63 [54–71]	59 [41–72]	64 [52–71]	59 [40–71]
Gender, female (%)	37 (35%)	19 [40%]	17 (29%)	26 (38%)	11 (31%)
LOS ICU	6.7 [2.7–15]	7.4 [3.3–13]	6.2 [2.3–16]	6.7 [2.8–12.9]	6.4 [2.2–19]
APACHE III	66 [49–81]	69 [56–81]	59 [46–80]	66 [49–78]	67 [53–81]
ICU mortality	19 (18%)	11 (23%)	8 (14%)	13 (20%)	6 (17%)
Hospital mortality	26 (25%)	13 (28%)	13 (22%)	19 (28%)	7 (19%)
Invasive ventilation	59 (56%)	26 (55%)	31 (53%)	31 (45%)	22 (61%)
P/F ratio	152 [122–258]	148 [111–202]	158 [132–319]	133 [111–154]	256 [174–360]
Inotropes	34 (32%)	16 (34%)	18 (31%)	17 (25%)	13 (36%)
DVT prophylaxis *	103 (98%)	46 (98%)	57 (98%)	67 (97%)	36 (100%)
*Primary diagnostic category*					
Respiratory					
COVID-19	45 (58%)	21 (45%)	26 (45%)	48 (69%)	0
Non-COVID-19	4 (4%)	2 (4%)	2 (3%)	0	5 (14%)
Cardiac	8 (8%)	3 (6%)	5 (9%)	3 (4%)	5 (14%)
Gastrointestinal	8 (8%)	3 (6%)	5 (9%)	3 (4%)	5 (14%)
Neurological	6 (6%)	3 (6%)	3 (5%)	3 (4%)	2 (6%)
Renal	5 (5%)	4 (9%)	1 (2%)	4 (6%)	1 (3%)
Sepsis	15 (14%)	7 (15%)	8 (14%)	3 (4%)	10 (28%)
Trauma	10 (10%)	2 (4%)	8 (14%)	2 (3%)	8 (22%)
Other	4 (4%)	2 (4%)	0	3 (4%)	0
*Coagulation variables*					
Platelet count	210 [148–304]	200 [164–253]	232 [133–321]	211 [158–310]	194 [133–291]
PT	13 [13–15]	13 [12–14]	14 [13–15]	13 [13–15]	14 [13–15]
aPTT	29 [26–34]	29 [25–33]	30 [27–34]	29 [26–33]	30 [27–34]
INR	1.2 [1.1–1.3]	1.1 [1.0–1.2]	1.2 [1.1–1.3]	1.1 [1.1–1.3]	1.2 [1.1–1.4]
Fibrinogen	5.0 [3.8–6.4]	4.7 [3.7–5.8]	5.3 [4.0–7.0]	5.1 [3.8–6.3]	4.5 [3.6–6.0]
*ClotPro variables*					
FIB-test A10	27 [21–31]	24 [18–27]	30 [26–32]	26 [20–30]	29 [22–32]
EX-test A10	65 [59–67]	63 [57–65]	67 [60–69]	65 [58–67]	66 [60–68]
Platelet A10	38 [34–40]	38 [35–41]	37 [34–39]	38 [35–39]	38 [33–40]
TPA-test MCF	44 [36–53]	38 [29–43]	52 [46–59]	42 [33–49]	47 [38–56]
TPA-test LT	308 [268–418]	263 [195–274]	398 [331–530]	302 [252–364]	338 [270–432]

Values are medians with interquartile ranges in [brackets] or counts with percentage in (parentheses)

*LOS ICU* length of stay in intensive care unit, *APACHE* acute physiology and chronic health evaluation, *ICU* intensive care unit, *P/F ratio* ratio between arterial partial pressure (P) of oxygen and fraction (F) inspired oxygen, *DVT* deep venous thrombosis, *COVID-19* coronavirus disease of 2019, *PT* prothrombin time, *aPTT* activated partial thromboplastin time, *INR* international normalised ratio, *FIB*-test A10 clot amplitude at 10 min, surrogate measure for functional fibrinogen, *EX-test A10* clot amplitude at 10 min for testing the extrinsic coagulation pathway, *Platelet A10* amplitude at 10 min for the calculated contribution of platelets, *TPA-test MCF* maximum clot firmness for the fibrinolysis activation test, *TPA-test LT* time to lysis down to 50% of the clot amplitude by the fibrinolysis activation test

*Includes prophylactic dosing of heparin (5000 units twice daily) or enoxaparin (40 mg daily) and/or calf compressors

**This group includes patients with an incidental finding of a positive SARS-Cov2 PCR test while this was not the primary reason for admission to ICU

When compared with the healthy controls (*n* = 20), the critically ill patients showed significantly increased [median difference 12.5 (95% CI 9–15) mm, *p* < 0.0001] fibrin clot amplitude (FIB-test A10), with 71 (68%) cases above the upper level of normal (ULN) (23 mm). No significant difference in the amplitude of the fibrin clot formed (FIB-test A10) was found between COVID-19 and non-COVID-19 cases (Fig. 1A), median difference 0.5[95% CI − 2 to 4] mm, *p* = 0.46. The EX-test A10 was increased in critically ill patients compared to healthy controls (median difference 9.5 [95% CI 5–10] mm, *p* < 0.0001) with no difference between the COVID-19 and non-COVID-19 patients (Fig. 1B), median difference 0.0 [95% CI − 1 to 3] mm, *p* = 0.41. The platelet contribution to the clot, platelet A10, was reduced in critically ill patients compared to healthy controls (median difference 4.0 [95% CI 3–6] mm, *p* < 0.0001). Again, no difference was found between COVID-19 and non-COVID-19 cases in the calculated platelet A10 (Fig. 1C) (median difference 0.0 [95% CI − 2 to 2] mm, *p* = 0.77), and no difference was found in the platelet count between the two patient groups (median difference 2.0 [95% CI − 47 to 45] × 10^9^/L, *p* = 0.90) (data not shown).

Minimal variation was seen in the TPA-test LT in healthy controls (Fig. 1D). In contrast, critically ill patients demonstrated substantial variation in the TPA-test LT with a significantly higher median than in healthy controls (median difference 122 [95% CI 103–176] s, *p* < 0.0001). The non-COVID patients had a significant but smaller increase in TPA-test LT than COVID-19 patients (median difference 43.5 s [95% CI 2–112] mm, *p* < 0.036) (Fig. 1D). In the COVID-19 population, 35 of 69 (51%) patients had a TPA-test LT of > 300 s, compared to 23 of 36 patients (64%) in the non-COVID-19 population.

**Fig. 2:**
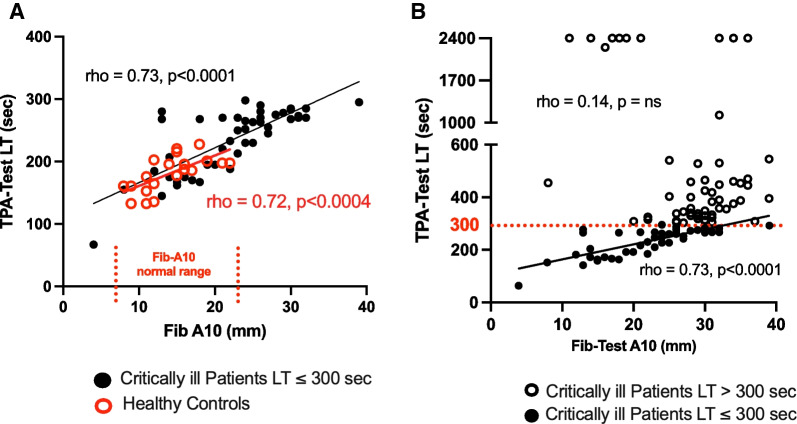
Fibrinolysis resistance measured by the TPA-test is not related to the volume of fibrin clot formed. **A** Correlation analyses between the fibrin clot amplitude (FIB-test A10) and the TPA-test LT in healthy controls (*n* = 20, red circles) and critically ill patients with a TPA-test LT ≤ 300 s (*n* = 47, black dots). Normal range for the FIB-test A10 shown between vertical red dotted lines and the lines of best fit (simple linear regression) provided for each group in the respective colours. **B** Correlation analyses between FIB-test A10 and TPA-test LT in critically ill patients with TPA-test LT ≤ 300 s (*n* = 47, black dots) and critically ill patients with TPA-test LT > 300 s (*n* = 58, open black circles) with red dotted line indicating cutoff of 300 s. Spearman’s correlation analysis (rho); ns = nonsignificant p value

To evaluate the impact of elevated fibrinogen levels on the TPA-test LT, correlation analyses were done with the FIB-test A10 as a surrogate fibrinogen measure in the combined critically ill population. In the healthy control population, the FIB-test A10 were all within the normal range (7–23 mm) and a correlation analysis between the FIB-test A10 and the TPA-test LT demonstrated a strong linear relationship (Fig. 2A) (rho = 0.72 [95% CI 0.39–0.88], *p* = 0.0004). Similar correlation analyses were conducted following division of the combined critically ill population based upon a TPA-test LT of ≤ 300 s or > 300 secs with the latter group considered to be fibrinolysis-resistant. In critically ill patients who were not fibrinolytic-resistant (*n* = 45; TPA-test LT ≤ 300 s), a strong correlation between the FIB-test A10 and the TPA-test LT was observed (Fig. 2A, B) (rho = 0.73 [95% CI 0.55–0.84], *p* > 0.0001). In contrast, correlation analysis between the FIB-test A10 and the TPA-test LT in fibrinolysis-resistant patients demonstrated no significant relationship (rho = 0.05 [95% CI − 2.44 to 0.29], *p* = 0.86) (Fig. 2B).

### Experimental t-PA and plasminogen supplemented ClotPro assays

**Fig. 3:**
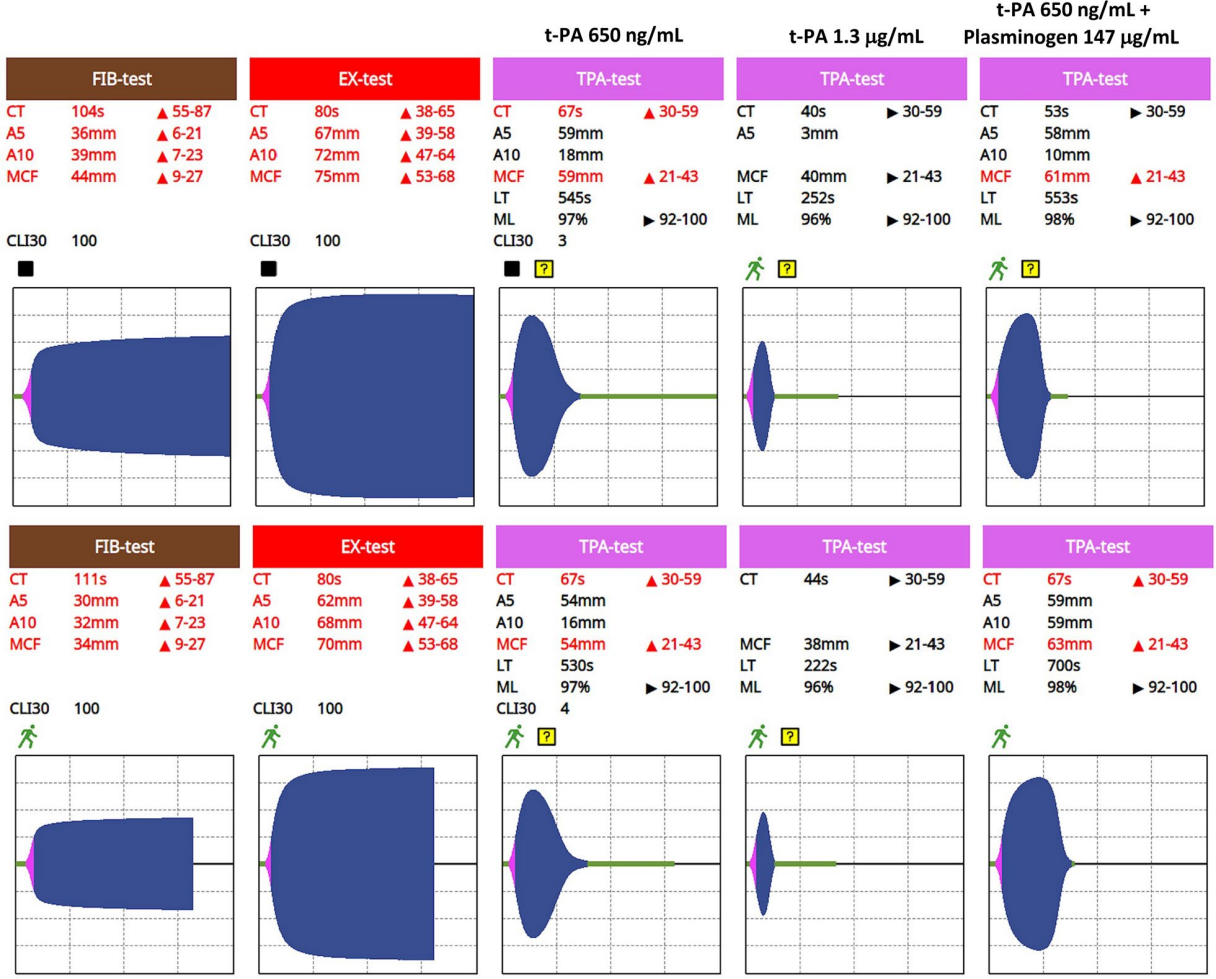
Examples of patients requiring an increased t-PA dose. Cases 15 and 16 (Additional file 1: Table S1) demonstrated above normal values in the clot amplitude (A5, A10) and maximum clot firmness (MCF) on both FIB-test and EX-test. The first of the TPA-tests was performed according to the manufacturer’s instructions and hence contained t-PA 650 ng/mL blood. In both cases, the LT was significantly prolonged at 545 and 530 s. In the second of the TPA-tests, the t-PA dose was doubled by loading the test according to the manufacturer’s instructions but passing the blood through a second TPA-test tip prior to commencing the assay. In both cases, the increased t-PA dose resulted in a significant shortening of the LT to that approximating the upper level seen in healthy controls. In the final TPA-tests on the right-hand side in each case, the blood was passed first through a tip containing plasminogen 147 μg/mL immediately prior to the TPA-test tip and the assay being commenced. In both cases, an increased MCF and prolonged LT were observed compared to the response to the single t-PA dose on its own

TPA-tests that were spiked with additional t-PA (total 1.3 μg/mL) were carriedout in 21 patients with a median reduction in LT of 200 [177–272] % seen in 17 (81%) cases (examples depicted in Fig. 3, Additional file 1: Table S1, Column B).

**Fig. 4:**
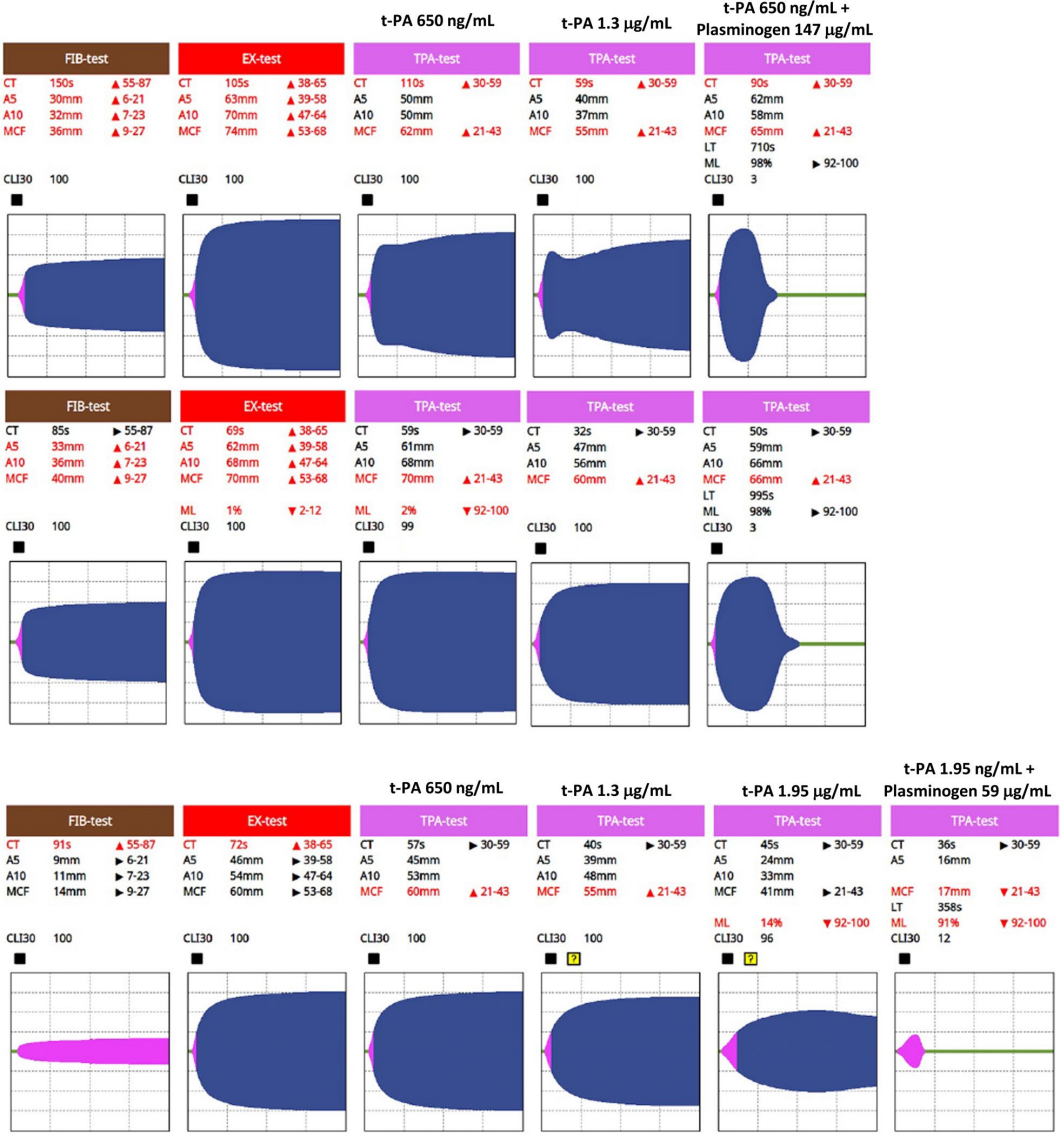
Example of patients requiring the combination of t-PA and plasminogen. Cases 27 and 28 (top two rows, Additional file 1: Table S1) demonstrated above normal values in the clot amplitude (A5, A10) and maximum clot firmness (MCF) on both FIB-test and EX-test. The first of the TPA-tests was performed according to the manufacturer’s instructions and hence contained t-PA 650 ng/mL blood. In both cases, little to no lysis occurred; hence, a LT was not registered. In the second of the TPA-tests, the t-PA dose was doubled as described in Fig. 3. In both cases, the increased t-PA dose did alter observed fibrinolysis and a LT was not registered. In the final TPA-tests on the right-hand side in cases 27 and 28, the blood was passed through a tip containing plasminogen 147 μg/mL as described in Fig. 3. In both cases, the addition of plasminogen induced clot lysis significantly shortening the LT which overall remained above normal. Case 5 (third row, Additional file 1: Table S1) demonstrates that on the standard TPA-test (650 ng/mL), the double-dose TPA-test (1.3 μg/mL) and even a triple-dose TPA-test (1.95 μg/mL), a concentration-dependent reduction in the MCF was observed but did not reach 50%, and therefore, no lysis time was registered. When plasminogen 59 μg/mL was added to the triple-dose TPA-test, a significant increase in clot lysis occurred and a LT was registered

The 4 (19%) cases who had no response to additional t-PA supplementation responded to plasminogen supplementation of the TPA-test resulting in a median 68.5 [61–81.3]% reduction in LT (examples depicted in Fig. 4).

Of the 23 cases who had plasminogen 147 μg/mL added to the TPA-test (Additional file 1: Table S1, Column D), 8 (35%) cases demonstrated a median reduction in LT of 69 [44–77]%. All these cases had a baseline TPA-test LT (Additional file 1: Table S1, Column A) of > 1000 s (Cases 1, 5, 10, 25–28, 32). Two of these cases (25 and 26) who registered a LT between 1000 and 2400 s, responded to additional t-PA supplementation (Additional file 1: Table S1, Column B), as well as to plasminogen supplementation of the TPA-test (Additional file 1: Table S1, Column D). In contrast, 15 (65%) cases demonstrated a median 51 [37–125]% *prolongation* of the lysis time in response to 147 μg/mL plasminogen supplementation (Additional file 1: Table S1, Column D). All these patients had a baseline TPA-test LT (Additional file 1: Table S1, Column A) of < 1000 s and all had responded to additional t-PA supplementation (Additional file 1: Table S1, Column B) when tested. Additional file 1: Fig. S2 illustrates the effect of plasminogen supplementation on the TPA-test LT in patients with a baseline TPA-test LT > 1000 s vs those with a LT < 1000 s. No such inhibitory effect on the LT was observed in 9 cases where the TPA-test was supplemented with the lower concentration of 59 μg/mL plasminogen (Additional file 1: Table S1, Column C) with reductions in LT observed irrespective of the baseline TPA-test LT (Additional file 1: Table S1, Column A); however, the reductions measured were not as substantial as those achieved with 147 μg/mL plasminogen, median 12 [7.5–32.5]%.

The addition of plasminogen (147 μg/mL) to ClotPro assays that do not contain exogenous t-PA (the EX-test ± FIB-test) was tested in 5 patients and resulted in changes of < 10% in the EX-test/FIB-test A10. A 20% reduction in EX-test A10 was seen in one individual who was receiving warfarin (data not shown).

To summarise the ex vivo supplementation data, two predominant groups emerged that were distinguished by the response to ex vivo fibrinolytic protein supplementation: (i) cases that responded to supplementation with t-PA alone, the majority of which had a baseline TPA-test LT of < 1000 s, and (ii) cases that responded to the combination of t-PA and plasminogen supplementation, the majority of which had a baseline TPA-test lysis times > 1000 s. Plasminogen supplementation in the absence of t-PA appeared ineffective at inducing significant clot lysis.

### Interventional study of ClotPro monitoring of 24 h alteplase infusion

**Fig. 5:**
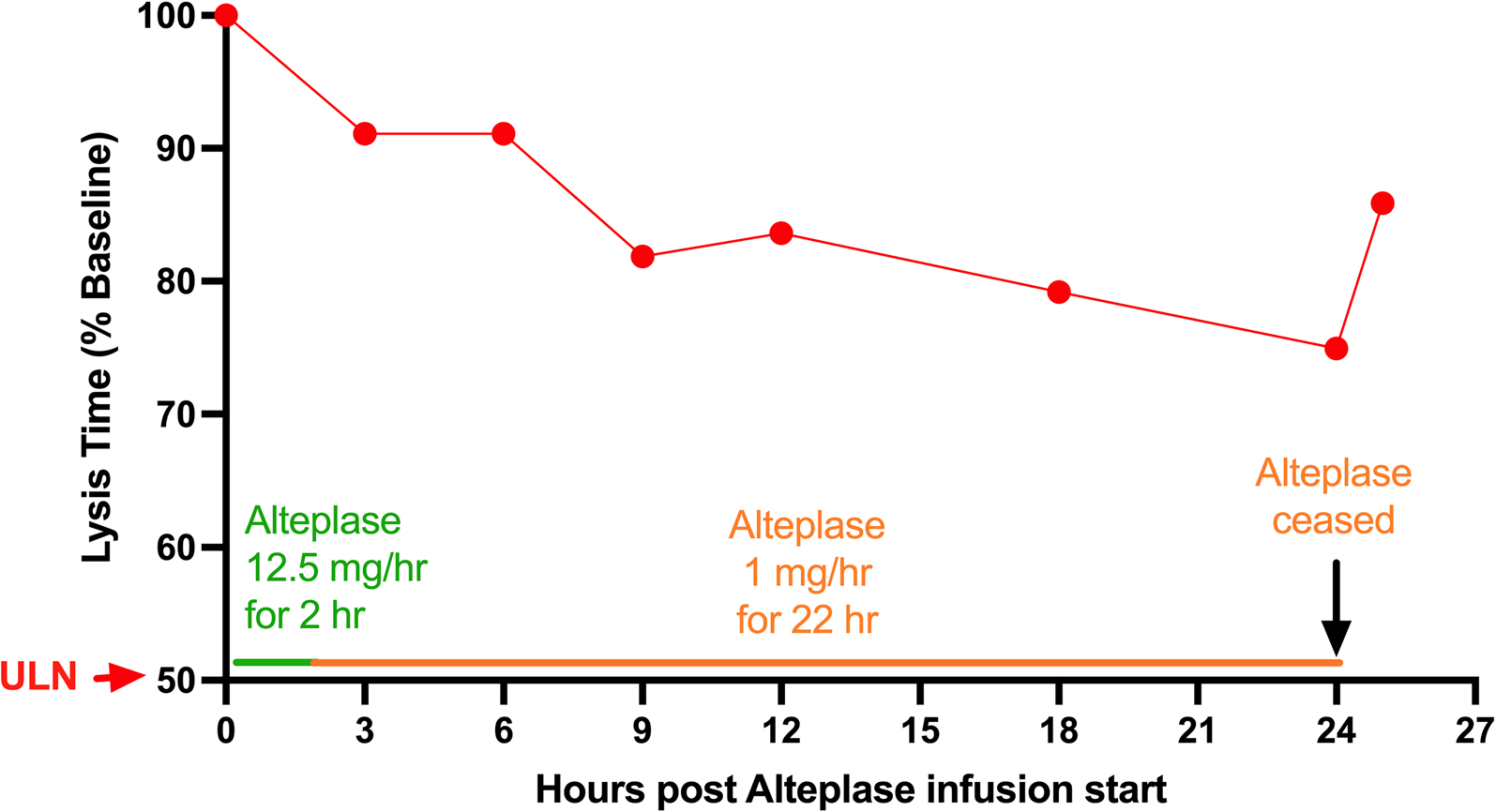
Monitoring changes in fibrinolysis during 24 h alteplase infusion. A patient with fibrinolysis resistance secondary to bacterial pneumonia complicated by ARF was identified using the ClotPro TPA-test. Baseline TPA-test LT was 450 s. A low-dose alteplase infusion was commenced starting with a 25 mg bolus over 2 h, followed by a 1 mg/h infusion over 22 h. ClotPro analysis was repeated at the times indicated with EX-test, FIB-test and TPA-tests being performed. Analyses were performed within 10 min of blood collection. The plot depicts the results of the TPA-test lysis time presented as a percentage of the baseline value. The upper level of normal (ULN) at 50% on the Y-axis approximates the highest level measured in our healthy control population (LT range: 132–227 s, *n* = 20)

Below are the results from a single patient who was enrolled in the safety and dose-finding stage of the Phase 2 safety, dose-finding and efficacy study evaluating viscoelastic testing (VET)-guided tissue plasminogen activator (t-PA) treatment in critically ill pro-thrombotic ARF (VETtiPAT-ARF) trial (NCT05540834). Clinical outcomes were not assessed in this stage of the study which had the primary intent of safety assessment. The patient enrolled had bacterial pneumonia causing ARF and fibrinolysis resistance (baseline TPA-test LT of 450 s). The lowest alteplase dosing regime (25 mg bolus of alteplase over 2 h, followed by 1 mg/hr for a further 22 h) was administered in keeping with the protocol. Frequent VET monitoring was performed throughout the infusion and within 10 min of blood collection. This revealed a gradual reduction in the TPA-test LT from the baseline TPA-test LT value of 450–336 s (25%) over 24 h. (A normal LT was not achieved in this patient with this low-dose regimen.) A partial relapse of 49 s in the LT was observed 1 h following cessation of the alteplase infusion (Fig. 5). No changes were observed in the EX-test and FIB-test parameters over the 24-h period (data not shown), and there were no signs of bleeding.

## Discussion

These data suggest that (i) fibrinolysis resistance is associated with many critical conditions requiring intensive care, and occurs with equal severity in COVID-19 and non-COVID-19 patients, (ii) a TPA-test lysis time of > 300 s appears to distinguish patients with fibrinolysis resistance from patients with slowed fibrinolysis due to elevated fibrinogen levels, (iii) ex vivo supplementation of t-PA on its own, or in combination with its substrate, plasminogen, is able to correct the majority of fibrinolysis-resistant cases, (iv) a TPA-test lysis time of > 1000 s appears to predict the beneficial response to combined t-PA and plasminogen supplementation, and (v) ClotPro VET and the associated TPA-test can potentially be used to monitor the response and guide the dose of in vivo t-PA supplementation that is delivered with the intention of restoring fibrinolysis.

In the normal healthy state, the fibrinolytic system is tightly regulated, as demonstrated by the low variation observed in our healthy control population. Significant recent attention has focused on fibrinolysis resistance that occurs in a proportion of patients with severe COVID-19 disease. Our study is a reminder that equally severe fibrinolysis resistance occurs in critically ill patients with a range of non-COVID-19-related diagnoses that have a systemic inflammatory response as a common feature.

The amplitude of the fibrin clot formed in the FIB-test closely correlates with plasma fibrinogen levels [40]. Correlation analyses between the amplitude of the fibrin clot (FIB-test A10) and the TPA-test LT in patients with a LT ≤ 300 s and those with a LT > 300 s (Fig. 2B) demonstrated that in patients with a LT > 300 s, the degree of fibrinolysis resistance was not due to the formation of larger fibrin clots, as was observed in healthy controls and patients with a LT ≤ 300 s (Fig. 2A). These data suggest that fibrinogen is the leading determinant of TPA-test lysis times when fibrinolysis is occurring normally; however, this relationship is lost, most likely due to a change in the balance of other factors, such as pro- and anti-fibrinolytic protein activity. The extent of fibrin cross-linking and the presence of neutrophil extracellular traps, bacterial or platelet polyphosphates, von Willebrand factor (vWF) and reactive oxygen species within the blood also negatively impact fibrinolysis [41–45].

The data obtained in the ex vivo supplementation experiments identified two states of fibrinolysis resistance: (i) one that could be corrected with t-PA supplementation alone, thus suggesting reduced t-PA activity such as due to an excess of PAI-1, and (ii) the other that required the addition of t-PA and plasminogen, thus suggesting reduced plasmin activity on its own, due to excessive consumption or inhibition by α2-antiplasmin, or in combination with reduced t-PA activity. As part of the acute phase inflammatory response, endothelial cells and platelets release t-PA and plasminogen activator-1 (PAI-1), the principal inhibitor of t-PA [46]. Thus, an imbalance of t-PA:PAI-1 levels may rapidly develop resulting in fibrinolysis resistance that correlates with multi-organ dysfunction syndrome and death in bacterial and viral infection [13, 47, 48]. Moreover, in a study of 29 patients critically ill with COVID-19, the ClotPro TPA-test LT was found to significantly correlate with plasma PAI-1 levels (*r* = 0.70; *p* < 0.0006) [27].

Reductions in plasmin activity also occur in systemic inflammation and may be due to insufficient t-PA to convert plasminogen to plasmin, high levels of plasmin inhibitors and/or plasmin consumption through intravascular fibrin degradation, tissue repair and several aspects of the immune response [39, 49]. In ARF patients, inhibition of plasmin activity was reported in bronchoalveolar lavage samples and was partially attributed to increased levels of α2-antiplasmin [50]. Additionally, in a study of patients with severe sepsis and septic shock, reduced levels of plasminogen and the coagulation inhibitors, anti-thrombin III (ATIII) and Protein C, were measured in comparison to patients with less severe sepsis, in whom fibrinolysis was strongly activated and coagulation inhibited by ATIII. The authors concluded that the consumption of plasminogen and coagulation inhibitors was the principal mechanism leading to fibrinolysis resistance in the more severely ill patients and they demonstrated that the same mechanisms occurred in sepsis due to several different pathogens [10]. Additional evidence for plasminogen consumption causing fibrinolysis resistance was obtained in burns patients with reduced plasminogen levels associating with the extent of burn injury and development of organ dysfunction [9]. Also contributing to depleted plasminogen levels is the finding that the protease released from activated neutrophils, neutrophil elastase, is capable of degrading plasminogen [51]. Several lines of evidence indicate, therefore, that the systemic inflammatory response following severe infection or injury maybe associated with fibrinolytic changes that are dynamic and characterised by early stage hyperfibrinolysis which transitions to fibrinolysis resistance due to factor consumption (Additional file 1: Fig. S3).

The ClotPro TPA-test and the novel exploratory extensions of this test described herein permit identification of fibrinolysis-resistant patients and potentially the corrective treatment required. The results of this study demonstrate the significant variation in the degree of fibrinolysis resistance that occurs between patients, as measured by the TPA-test LT, and in the amounts of t-PA ± plasminogen required to restore fibrinolysis ex vivo. These data imply that a personalised approach to the correction of fibrinolysis resistance is required rather than uniform protocols.

The dichotomous response to plasminogen supplementation may reflect endogenous plasminogen levels. A baseline LT > 1000 s may indicate reduced plasminogen levels; thus, supplementation in the presence of t-PA enhanced clot lysis. In contrast, where the baseline LT is < 1000 s, supplemented plasminogen combined with sufficient endogenous levels may result in the inhibition of clot lysis. We are unaware of this effect being previously described, although a previous study demonstrated inhibitory effects of high t-PA levels on plasmin lysis of fibrin [52]. Further ex vivo experiments are planned to correlate viscoelastometry results with laboratory-based measures of fibrinolytic proteins and to investigate the mechanism of the observed inhibitory effect.

The utility of the ClotPro TPA-test to monitor in real time the effect on fibrinolysis of systemic fibrinolytic protein administration, was demonstrated, in this case with alteplase. The working hypothesis for the VETtiPAT-ARF trial (NCT05540834) is that the infusion of low-dose t-PA over days will correct reduced t-PA activity resulting in the restoration of fibrinolysis but without compromising coagulation. The unchanged EX-test and FIB-test parameters measured during the alteplase infusion demonstrated that coagulation in response to tissue factor was preserved throughout. A similar method could also be used to monitor plasminogen administration or the combination of both proteins. This novel method mimics the use of activated partial thromboplastin clotting time (aPTT) to monitor therapeutic heparin administration, with the contemporary capacity to dose the supplemented fibrinolytic protein/s according to the TPA-test LT. Additionally, this approach enables the dose of the fibrinolytic protein to be tailored to the patient’s requirements, thus potentially increasing efficacy while reducing thrombosis and bleeding risk, as well as cost.

## Study limitations

We did not attempt to correlate the extent of fibrinolysis resistance with clinical outcomes, for example thromboembolic complications, as this would require a much larger study. The definition of fibrinolysis resistance used in this study (TPA-test LT of > 300 s) would also need to be correlated with clinical outcomes in such studies. A single point-of-care technology was used in this study to rapidly evaluate fibrinolysis resistance in whole blood. There is currently no gold standard to diagnosis fibrinolysis resistance, and no alternative technology that could have been used to correlate the ClotPro results was available to us. This study was commenced during the second wave of the COVID-19 pandemic in Australia. The local Ethics Committee provided expedited approval for observational studies, but the waiver of informed consent did not allow for the collection, storage and later analysis of plasma samples for fibrinolytic protein analysis. Future studies are planned to correlate the ClotPro TPA-test parameters with plasma concentrations of fibrinolysis-associated proteins.

## Conclusions

This study has demonstrated the potential of the ClotPro device to identify and correct fibrinolysis resistance in critically ill patients. Two response groups were identified: (i) those requiring t-PA supplementation alone and (ii) those requiring both t-PA and plasminogen supplementation, with the TPA-test lysis time, a measure of the extent of fibrinolysis resistance, potentially delineating these groups. The use of this methodology may enable more rapid and targeted intervention to restore fibrinolysis and preserve organ function. Additionally, this point-of-care technology enables close monitoring of the response to in vivo fibrinolysis protein supplementation, thus permitting dose optimisation at an individual level which will potentially translate into risk reduction and improved patient outcomes. These methods and results may have far reaching therapeutic implications for many critical conditions associated with compromised fibrinolysis.

## Supplementary Information

**Additional file 1. Supplementary Figure 1**. Flowchart of Patient Numbers Included in Experiments.** Supplementary Table 1**. Effect of ex-vivo t-PA +/- plasminogen supplementation on clot lysis times and plasminogen on clot amplitude.** Supplementary Figure 2**. Effect of plasminogen supplementation on TPA-test lysis time is dependent upon the degree of fibrinolysis resistance present, as reflected in the baseline TPA-test LT.** Supplementary Figure 3**. The acute phase response (APR), following isolated or severe injury or infection, and the utility of ClotPro VET to guide fibrinolytic protein supplementation to correct fibrinolysis resistance.

## Data Availability

Data are provided in the manuscript. If any additional information is required, email l.coupland@unsw.edu.au.

## Abbreviations

ARDS: Acute respiratory distress syndrome
CI: Confidence interval
COVID-19: Coronavirus disease-19
LT: Lysis time
MODS: Multi-organ dysfunction syndrome
PAI-1: Plasminogen activator inhibitor-1
SD: Standard deviation
t-PA: Tissue plasminogen activator
VET: Viscoelastic testing
