# Supplementary material for: Point-of-care diagnosis and monitoring of fibrinolysis resistance in the critically ill: results from a feasibility study
Source: Crit Care. 2023 Feb 10;27:55. doi: 10.1186/s13054-023-04329-5 (PMC9912243; doi:10.1186/s13054-023-04329-5)
Supplement: Supplementary file 1 — Additional file 1. Supplementary Figure 1. Flowchart of Patient Numbers Included in Experiments. Supplementary Table 1. Effect of ex-vivo t-PA +/- plasminogen supplementation on clot lysis times and plasminogen on clot amplitude. Supplementary Figure 2. Effect of plasminogen supplementation on TPA-test lysis time is dependent upon the degree of fibrinolysis resistance present, as reflected in the baseline TPA-test LT. Supplementary Figure 3. The acute phase response (APR), following isolated or severe injury or infection, and the utility of ClotPro VET to guide fibrinolytic protein supplementation to correct fibrinolysis resistance. [file 13054_2023_4329_MOESM1_ESM.docx]

**Supplementary Figure 1: Flowchart of Patient Numbers Included in Experiments**

| **Case**  **No.** | **COVID-19**  **Status**  **(PCR)** | **Principal**  **Diagnosis** | ***A***  **TPA-test^#^**  (t-PA 650 ng/ mL)  (LT sec) | ***B***  **TPA-test x 2**  (t-PA 1.3 μg/mL)  (LT sec) | ***C***  **TPA-test**  **+ 59 μg Plg/mL**  (LT sec) | ***D***  **TPA-test**  **+ 147 μg Plg/mL**  (LT sec) |
| --- | --- | --- | --- | --- | --- | --- |
| 1 | Positive | Bacterial pneumonia | >2400 |  |  | 290 (-88%) |
| 2 | Positive | Sub-arachnoid haemorrhage | 383 | 175 (-44%) |  |  |
| 3 | Positive | Bacterial pneumonia | 453 |  |  | 713 (+57%) |
| 4 | Positive | Pneumonitis | 338 | 185 (-45%) | 308 (-9%) |  |
| 5 (Fig 4) | Positive | Pneumonitis | >2400 | >2400 (0%) | - | 358 (-85%)  *NB: 1.95 μg/mL TPA* |
| 6 | Positive | Pneumonitis | >2400 | 305 (-87%) | 2400 (0%) |  |
| 7 | Positive | Pneumonitis | 415 |  | 368 (-11%) |  |
| 8 | Positive | Pneumonitis | 403 |  | 172 (-57%) |  |
| 9 | Positive | Diarrhoea, septic shock | 450 |  | 398 (-12%) |  |
| 10 | Positive | Hypovolaemic shock – bleed | >2400 |  | 2247 (-6%) | 668 (-72%) |
| 11 | Positive | Pneumonitis | 460 |  | 370 (-20%) |  |
| 12 | Positive | Pneumonitis | 465 | 288 (-38%) | 298 (-36%) |  |
| 13 | Negative | Bacterial pneumonia, ARF | 450 |  |  | 655 (+46%) |
| 14 | Negative | Sepsis, MODS | 340 |  | 240 (-29%) |  |
| 15 (Fig 3) | Negative | Hypoxic arrest, endocarditis | 530 | 222 (-58%) |  | 700 (+32%) |
| 16 (Fig 3) | Negative | Acute severe pancreatitis | 545 | 252 (-54%) |  | 553 (+1%) |
| 17 | Negative | Ischaemic bowel | 348 | 167 (-52%) |  | 990 (+184%) |
| 18 | Negative | Severe sepsis, MODS | 425 | 190 (-55%) |  | 580 (+37%) |
| 19 | Negative | Polytrauma, sepsis | 425 | 177 (-58%) |  | 623 (+47%) |
| 20 | Negative | Necrotising fasciitis | 690 |  |  | 813 (+18%) |
| 21 | Negative | STEMI, necrotising fasciitis | 373 |  |  | 558 (+50%) |
| 22 | Negative | Trauma, multi DVTs | 330 | 157 (-52%) |  | 568 (+72%) |
| 23 | Negative | Sepsis, foot amputation | 390 | 200 (-49%) |  | 588 (+51%) |
| 24 | Negative | Polytrauma, sepsis | 388 | 192 (-51%) |  | 633 (+63%) |
| 25 | Negative | Polytrauma | 1125 | 425 (-62%) |  | 685 (-39%) |
| 26 | Negative | SIRS post cardiac bypass | 2107 | 578 (-73%) |  | 465 (-78%) |
| 27 (Fig 4) | Negative | Saddle PE, Influenza ARF | 2400 | 2400 (0%) |  | 710 (-70%) |
| 28 (Fig 4) | Negative | Necrotising cholecystitis | >2400 | 2400 (0%) |  | 995 (-59%) |
| 29 | Negative | Severe pancreatitis | 325 | 200 (-39%) |  | 760 (+233%) |
| 30 | Negative | Multiple trauma exc. head | 345 | 177 (-49%) |  | 605 (+175%) |
| 31 | Negative | Multiple trauma exc. head | 465 | 255 (-45%) |  | 580 (+125%) |
| 32 | Negative | Multiple trauma exc. head | >2400 | 2400 (0%) |  | 793 (-67%) |

**Supplementary Table 1: Effect of ex-vivo t-PA +/- plasminogen supplementation on clot lysis times and plasminogen on clot amplitude.**

# baseline value against which other TPA-test results are compared with % change from # value provided in brackets; LT = lysis time; Plg = Plasminogen; A10 = Clot amplitude at 10 min; ARF = Acute respiratory failure, MODS – multi-organ dysfunction syndrome, PE – pulmonary embolism, SIRS – systemic inflammatory response syndrome, DVT – deep vein thrombosis.

**Supplementary Figure 2: Effect of plasminogen supplementation on TPA-test lysis time is dependent upon the degree of fibrinolysis resistance present, as reflected in the baseline TPA-test LT**. A comparison was made of the effect of plasminogen supplementation on the TPA-test lysis time (LT) between patients who had a baseline TPA-test LT of <1000 seconds (s) (n=15) and patients who had a baseline TPA-test LT of >1000 s (n=8). The difference between the baseline TPA-test LT and the plasminogen supplemented TPA-test LT is expressed as % change with a negative value indicating a shortening of the lysis time. Individual data points presented, bars represent median and interquartile range.

**Supplementary Figure 3. The acute phase response (APR), following isolated or severe injury or infection, and the utility of ClotPro VET to guide fibrinolytic protein supplementation to correct fibrinolysis resistance.**

**A.** After an isolated injury or infection, the APR follows a predictable and quantifiable time-course with minimal risk of complications. The biological systems activated during the APR rapidly change and can be generally divided into two biologically distinct phases: ‘survival-APR’ and ‘repair-APR’. Temporally, survival-APR precedes repair-APR, occurring during early convalescence. It involves activation of hemostasis (a) and survival inflammatory cells (b), which work together to activate the serine protease thrombin that, in its canonical role, activates platelets and converts fibrinogen to fibrin. Together the fibrin platelet plug contains ruptured compartments, both intravascular and extravascular and prevents, or mitigates, invasion by pathogens (c). Once containment is achieved, the survival-APR transitions to repair-APR (indicated by circular arrows). In this phase, the serine protease plasmin (d) promotes repair – in its canonical role to remove fibrin (fibrinolysis). Additionally, in its non-canonical role it is used and activated by reparative inflammatory cells to degrade and remove damaged tissues, either directly or indirectly promotes angiogenesis, and tissue reconstruction by activating growth factors and other proteases (e.g. MMPs) from their precursor forms and egress of mesenchymal stem cells. **B.** After severe injury, e.g., Level-1 trauma, or with sepsis, the APR follows a less predictable time-course with great potential for adverse outcomes. A key biological event portending mortality in these cases are either early hyperfibrinolysis (a) or later fibrinolysis resistance (b). Both of these states can be detected using point-of-care VET. Early hyperfibrinolysis is associated with severe bleeding and vascular leak and is a major cause of death. Later fibrinolysis resistance may be caused by multiple mechanisms (see text) and is associated with macro and micro thrombosis and multi-organ dysfunction syndrome (MODS) and is also a major cause of death. **C.** Here we demonstrate the utility of the ClotPro test to rapidly detect fibrinolysis resistance, determine ex-vivo if the addition of t-PA and/or Plasminogen (PLG) are sufficient to restore fibrinolysis, and to monitor in-vivo supplementation of fibrinolysis proteins to reverse the prolonged fibrinolysis resistance state, reducing the risk of multi-organ dysfunction and death.
